# Development and validation of a machine learning model to predict comorbid hypertension in patients with type 2 diabetes

**DOI:** 10.3389/fmed.2026.1754916

**Published:** 2026-02-18

**Authors:** Hailu Yang, Changfeng Fan, Chunyan Liu

**Affiliations:** 1 Department of Internal Medicine, Third People's Hospital of Lishui District, Nanjing, China; 2 Department of Thoracic Surgery, Nanjing Yimin Hospital, Nanjing, China; 3 Huaqiao Road Community Health Service Center, Nanjing, China

**Keywords:** hypertension risk, machine learning, predictive modeling, random forest, type 2 diabetes mellitus

## Abstract

**Background:**

Hypertension is a critical comorbidity in patients with type 2 diabetes mellitus that significantly increases cardiovascular risk. Although several predictive models have been developed using conventional logistic regression or basic machine learning algorithms, these approaches often face significant limitations. Many existing models suffer from a lack of external validation which limits their generalizability, or they operate as black boxes without providing interpretable clinical insights. Furthermore, most prior studies have focused exclusively on biological indicators while overlooking the potential impact of socioeconomic determinants and lifestyle factors on disease progression.

**Objective:**

To address these gaps, this study aimed to develop a high-performance Random Forest model for predicting hypertension risk in diabetic patients by integrating multidimensional data, including clinical metrics, lifestyle habits, and socioeconomic status. The study further sought to validate the model's robustness using an independent external cohort and assess its clinical utility through SHAP analysis, providing transparent interpretations of risk factors to guide personalized medical decision-making.

**Methods:**

A multicenter retrospective cohort study was conducted using electronic medical records from two tertiary hospitals. Eligible adults with type 2 diabetes and no prior hypertension were included. A total of 900 eligible patients were included, with 420, 180, and 300 participants in the training, testing, and external validation cohorts, respectively. Feature selection combined Boruta and LASSO methods, yielding seven predictors. Seven algorithms were tested, and model performance was assessed through cross-validation, independent testing, and external validation. The random forest model was explained using SHAP analysis.

**Results:**

Among 900 participants, the random forest model achieved the best discrimination, with AUCs of 0.89 in internal testing and 0.83 in external validation. Calibration and decision curve analyses confirmed stability and clinical utility. Key predictors included alcohol consumption, triglycerides, diabetes duration, health insurance type, fasting blood glucose, estimated glomerular filtration rate, and exercise frequency.

**Conclusion:**

The validated random forest model effectively predicts hypertension in type 2 diabetes patients, integrating metabolic, behavioral, and socioeconomic factors. Its interpretability and robust performance support its potential use for early identification and personalized prevention of hypertension in clinical practice.

## Introduction

Type 2 diabetes mellitus (T2DM) and hypertension are two of the most prevalent chronic conditions worldwide, often coexisting and exerting synergistic effects on cardiovascular morbidity and mortality (1, 2). Hypertension develops in a substantial proportion of individuals with T2DM, accelerating microvascular and macrovascular complications such as nephropathy, retinopathy, and atherosclerotic cardiovascular disease (3). Early identification of patients at high risk for developing hypertension after diabetes onset is therefore essential for timely intervention and prevention of adverse outcomes (4).

Traditional statistical approaches have provided valuable insights into risk factors for comorbid hypertension, including age, obesity, dyslipidemia, and poor glycemic control (5). However, these models often assume linear relationships and may fail to capture complex interactions among clinical, biochemical, and behavioral variables (6). In recent years, machine learning techniques have emerged as powerful tools to model nonlinear patterns and integrate multidimensional data, offering improved predictive accuracy and potential for individualized risk stratification (7, 8).

Despite this promise, few predictive models for hypertension in patients with T2DM have undergone rigorous external validation or incorporated interpretability to support clinical use. The capacity to understand model predictions is crucial for ensuring transparency and trust in algorithm assisted decision making.

Accordingly, this study aimed to develop and externally validate an interpretable machine learning model using routine clinical data to predict comorbid hypertension in patients with T2DM. Through systematic feature selection, model comparison, and explainable analysis, the work seeks to provide a robust, transparent tool for early risk identification and personalized prevention in clinical practice.

## Materials and methods

### Study design and participants

In this multicenter, retrospective study, we initially screened the medical records of 1,150 patients with type 2 diabetes admitted to the Third People's Hospital of Lishui District and Huaqiao Road Community Health Service Center between January 1, 2020, and May 31, 2025.

To ensure the validity of the analysis, strict exclusion criteria were applied. We excluded participants based on the following reasons: (1) documented hypertension prior to the diagnosis of diabetes (*n* = 108); (2) undetermined key predictors or outcome variables (*n* = 65); (3) missing or inconsistent dates regarding the diagnosis of diabetes or hypertension (*n* = 52); (4) diagnosis of type 1 diabetes, gestational diabetes, or pregnancy (*n* = 18); and (5) duplicate or clearly erroneous records (*n* = 7).

After excluding 250 ineligible participants, a total of 900 patients were included in the final analysis. The cohort from the Third People's Hospital of Lishui District (*n* = 600) was randomly divided into a training cohort (*n* = 420) and a testing cohort (*n* = 180) at a 7:3 ratio. The cohort from Huaqiao Road Community Health Service Center (*n* = 300) served as the independent external validation set. The detailed selection process is presented in the flow diagram (Supplementary Figure S1).

We used electronic medical records from two tertiary hospitals. The index date for each patient was the date of the confirmed diagnosis of type 2 diabetes. All variables were identified in relation to this index date to keep a clear time order. Hypertension information was collected only after the diagnosis of type 2 diabetes. Participants were included if they were adults with a confirmed diagnosis date of type 2 diabetes, had at least one visit after that date where blood pressure or antihypertensive medication use was recorded, and had the key baseline variables available at or before the index date. The primary outcome of this study was the incidence of new-onset hypertension. To ensure clinical applicability and standardized interpretation, the prediction window was defined as the development of hypertension within 3 years following the initial diagnosis of T2DM. Hypertension was defined according to the 2024 ADA Standards of Care and Chinese Guidelines for Prevention and Treatment of Hypertension: systolic blood pressure ≥140 mmHg and/or diastolic blood pressure ≥90 mmHg on at least two occasions on different days, or the initiation of antihypertensive medication.

To address missing data and preserve the sample size for model training, we employed a data imputation strategy. First, variables with a missing data rate exceeding 20% were excluded from the dataset to avoid introducing significant noise. For the remaining variables, missing values were handled based on data type: continuous variables were imputed using the mean of the available data, and categorical variables were imputed using the mode. This approach allowed us to retain a maximal number of patient records while maintaining the statistical integrity of the dataset.

The primary outcome was comorbid hypertension occurring after the diagnosis of type 2 diabetes. We identified it from diagnosis codes and discharge summaries and confirmed it using blood pressure readings or antihypertensive prescriptions, requiring the first qualifying record to appear after the index date. Predictors were extracted at the index visit or within a short window around it, and for repeated measures we used the value closest to the index date and strictly before the first hypertension record to avoid information leakage. Predictors covered simple demographics, lifestyle factors, medical history related to diabetes, anthropometrics, and routine laboratory tests, with units harmonized across hospitals. All definitions and extraction rules were finalized before model building, and the testing and external validation cohorts were kept untouched until the final evaluation. Among all patients who developed hypertension, the median follow-up time from T2DM diagnosis to the onset of hypertension was 3.5 years, with an interquartile range of 2.8–5.7 years.

### Feature selection

To prevent information leakage and ensure unbiased performance evaluation, data partitioning was performed prior to any feature selection process. The dataset was randomly split into a training set (70%) and a testing set (30%). Feature selection techniques (LASSO and Boruta) were subsequently applied exclusively to the training set to identify optimal predictors. The testing set was strictly reserved for final model evaluation and was not involved in the feature selection or model training phases. We began with all candidate variables collected in the study, including demographics, lifestyle factors, clinical history, and laboratory tests. Feature selection was carried out only in the training cohort to avoid information leakage. Categorical variables were expanded into one-hot indicators and continuous variables were standardized for algorithms that require scaling. We used two complementary methods so that signals supported by different modeling assumptions could be captured. First, we applied the Boruta wrapper built on random forests, which compares the importance of each real variable with that of shuffled “shadow” variables and keeps a variable only when its importance is consistently higher than the shadow distribution. In parallel, we fitted a LASSO logistic model to perform shrinkage and selection; the penalty parameter λ was chosen by stratified five-fold cross-validation using binomial deviance as the criterion. After both procedures were completed, we defined the final predictor set as the intersection of the variables retained by Boruta and by LASSO. This conservative rule reduces redundancy and favors stable, reproducible predictors that are relevant for both tree-based and linear sparse models. The selected features were then carried forward unchanged to build and compare all machine learning models, while the testing and external validation cohorts remained untouched until final evaluation.

### Model construction and comparison

Following feature selection, only the seven consensus predictors retained by both Boruta and LASSO (Alcohol consumption, Triglycerides, Diabetes duration, Health insurance type, Fasting blood glucose, estimated glomerular filtration rate, and Exercise frequency) were used to build all models. We developed seven candidate classifiers on the training cohort: decision tree, k nearest neighbors, light gradient boosting machine, naïve Bayes, random forest, support vector machine, and extreme gradient boosting.

Preprocessing and resampling were performed strictly within the training folds to prevent information leakage. Categorical variables were one hot encoded. Continuous variables were standardized for algorithms sensitive to scaling, including support vector machine, k nearest neighbors, and naïve Bayes. Stratified five fold cross validation preserved the outcome prevalence in each fold.

Hyperparameters for each algorithm were tuned within the cross-validation framework using grid or randomized search. The specific list of hyperparameters, their search ranges, and the final optimal settings for each algorithm are detailed in Supplementary Table S1. Model selection prioritized the mean cross validated area under the receiver operating characteristic curve as the primary performance criterion, with accuracy, sensitivity, specificity, positive predictive value, negative predictive value, precision, recall, and F1 score as secondary measures. After tuning, the final instance of each algorithm was refit on the full training cohort with the selected hyperparameters.

Comparative performance was summarized by the cross validated area under the receiver operating characteristic curve and its confidence interval. Pairwise differences in area under the receiver operating characteristic curves between classifiers were assessed using the DeLong method with multiplicity adjustment. For the model chosen as primary, an operating threshold was determined by maximizing Youden's index on the training data, and the corresponding confusion matrix was used to report threshold dependent metrics.

Generalizability was assessed in two stages. First, all trained models were evaluated on the independent testing set. Second, the selected model was assessed in the external validation cohort. For both datasets, we reported area under the receiver operating characteristic curve with confidence intervals and summarized threshold dependent metrics at the prespecified operating point. Calibration was examined using flexible reliability curves together with calibration intercept and slope; clinical utility was appraised by decision curve analysis across a clinically relevant range of threshold probabilities.

### The SHAP to model interpretation

We used SHapley Additive exPlanations (SHAP) to quantify feature contributions to the model output at both global and individual levels, ensuring local accuracy and consistency. Explanations were generated for the final tree ensemble model using TreeExplainer on the independent testing set and replicated in the external validation set to avoid information leakage. The background distribution was derived from the training cohort using a compact representative subset to approximate the joint feature distribution. SHAP values were computed on the log-odds scale and, when needed, mapped to probability for interpretation. Global importance was summarized by the mean absolute SHAP value per feature. One-hot encoded categories were aggregated back to their parent variable to reflect clinical constructs.

### Statistical analyses

All statistical analyses and data visualizations were performed using R (version 4.4.2) and JD_DCPM (V6.11, Jingding Medical Technology Co., Ltd.). Continuous variables were assessed for normality via the Shapiro-Wilk test. Normally distributed data are presented as mean ± standard deviation, with group comparisons conducted using Student's *t*-tests. Non-normally distributed variables are expressed as median and interquartile range [M (Q1, Q3)] and analyzed via the Mann-Whitney *U* test. Categorical variables are reported as frequencies (percentages) and evaluated using Chi-square tests or Fisher's exact tests (for cell counts < 5). Statistical significance was defined as a two-tailed *P*-value < 0.05. The optimal cut-off value for the predictive model was determined using Youden's index, which maximizes the sum of sensitivity and specificity. Clinically, this metric is particularly relevant as it provides a balanced threshold that weighs the importance of correctly identifying high-risk patients against the need to minimize false positives. This balance is essential in clinical practice to ensure that preventative interventions are targeted effectively without causing unnecessary burden on patients or healthcare resources.

## Result

### Patient characteristics for training, testing, and external validation cohorts

A total of 900 participants were included in the study, comprising 420 in the training cohort, 180 in the testing cohort, and 300 in the external validation cohort. The baseline characteristics were well-balanced across the three groups. As detailed in Table 1, there were no statistically significant differences in demographic factors (age, sex, BMI), socioeconomic status (education, employment, insurance type), or lifestyle habits (alcohol consumption, smoking, exercise frequency) among the cohorts (all *P* > 0.05). Similarly, clinical measurements including diabetes duration, lipid profiles (Triglycerides, TC, LDL-C, HDL-C), fasting blood glucose, and renal function (eGFR, serum creatinine) showed comparable distributions across the training, testing, and validation sets (all *P* > 0.05). These results confirm that the cohorts are statistically comparable for model development and validation.

**Table 1:** Baseline characteristics of the training, testing, and external validation sets.

**Characteristic**	**Training cohort *N* = 420**	**Testing cohort *N* = 180**	**Validation cohort *N* = 300**	***P*-value**
Age (years), mean ± SD	58.9 ± 10.6	61.4 ± 9.8	60.1 ± 10.7	0.18
**Sex**, ***n*** **(%)**				
Male	205 (48.8)	86 (47.8)	148 (49.3)	0.78
Female	215 (51.2)	94 (52.2)	152 (50.7)	
**Education level**, ***n*** **(%)**				
Primary school or below	90 (21.4)	40 (22.2)	64 (21.3)	0.24
Junior high school	150 (35.7)	65 (36.1)	110 (36.7)	
Senior high school	110 (26.2)	45 (25.0)	80 (26.7)	
College or above	70 (16.7)	30 (16.7)	46 (15.3)	
BMI (kg/m^2^), mean ± SD	27.0 ± 3.8	25.8 ± 3.6	26.5 ± 3.9	0.14
**Occupation**, ***n*** **(%)**				
No	200 (47.6)	88 (48.9)	144 (48.0)	0.62
Yes	220 (52.4)	92 (51.1)	156 (52.0)	
**Health insurance type**, ***n*** **(%)**				
URBMI	244 (58.1)	108 (60.0)	168 (56.0)	0.33
UEBMI	176 (41.9)	72 (40.0)	132 (44.0)	
**Alcohol consumption**, ***n*** **(%)**				
Never	265 (63.1)	110 (61.1)	188 (62.7)	0.11
Previous/current	155 (36.9)	70 (38.9)	112 (37.3)	
**Smoking**, ***n*** **(%)**				
Never	300 (71.4)	126 (70.0)	214 (71.3)	0.16
Previous/current	120 (28.6)	54 (30.0)	86 (28.7)	
**Exercise frequency**, ***n*** **(%)**				
Never	92 (21.9)	38 (21.1)	64 (21.3)	0.29
Occasionally	160 (38.1)	70 (38.9)	116 (38.7)	
More than once per week	110 (26.2)	48 (26.7)	78 (26.0)	
Daily	58 (13.8)	24 (13.3)	42 (14.0)	
Diabetes duration (years), M (Q1, Q3)	8.2 (3.5, 12.8)	7.9 (3.2, 12.4)	8.5 (3.6, 13.0)	0.48
WHtR, M (Q1, Q3)	0.57 (0.53, 0.62)	0.56 (0.52, 0.61)	0.57 (0.53, 0.62)	0.36
Triglycerides (mmol/L), M (Q1, Q3)	1.82 (1.23, 2.49)	1.76 (1.17, 2.42)	1.88 (1.25, 2.55)	0.32
Fasting blood glucose (mmol/L), M (Q1, Q3)	7.7 (6.6, 9.0)	7.5 (6.4, 8.8)	7.6 (6.5, 8.9)	0.44
eGFR (ml·min^−1^·1.73 m^−2^), M (Q1, Q3)	88.5 (79.0, 98.6)	89.2 (79.8, 99.0)	87.8 (78.3, 97.5)	0.57
Total cholesterol (mmol/L), M (Q1, Q3)	4.62 (4.01, 5.21)	4.55 (3.98, 5.18)	4.66 (4.05, 5.24)	0.22
HDL-C (mmol/L), M (Q1, Q3)	1.10 (0.95, 1.28)	1.09 (0.94, 1.27)	1.11 (0.96, 1.29)	0.38
LDL-C (mmol/L), M (Q1, Q3)	2.72 (2.18, 3.23)	2.66 (2.14, 3.18)	2.74 (2.20, 3.25)	0.27
Serum creatinine (mg/dl), M (Q1, Q3)	0.91 (0.78, 1.05)	0.90 (0.77, 1.03)	0.92 (0.79, 1.06)	0.53
Osteoporosis, *n* (%)	48 (11.4)	20 (11.1)	34 (11.3)	0.41
Comorbid hypertension, *n* (%)	86 (20.5)	36 (20.0)	61 (20.3)	0.71

Data are presented as n (%) for categorical variables and as mean ± SD or median (IQR) for continuous variables, as appropriate. The symbol (%) denotes the percentage of patients within the corresponding cohort.

BMI, body mass index; URBMI, Urban Resident Basic Medical Insurance; UEBMI, Urban Employee Basic Medical Insurance; WHtR, waist-to-height ratio; eGFR, estimated glomerular filtration rate; HDL-C, high-density lipoprotein cholesterol; LDL-C, low-density lipoprotein cholesterol.

### Feature selection

**Figure 1:**
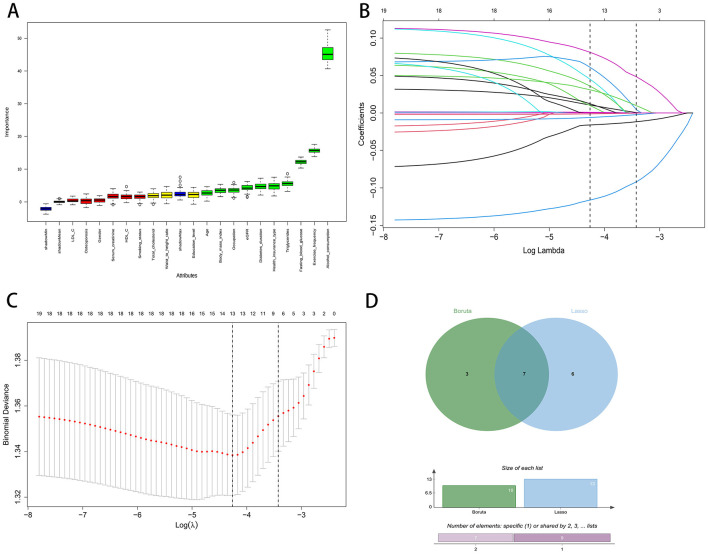
Feature selection process using Boruta and LASSO regression. **(A)** Boruta variable importance plot. **(B)** LASSO coefficient paths. **(C)** LASSO cross-validation curve. **(D)** Venn diagram of Boruta and LASSO.

Using the Boruta wrapper based on random forests, we identified 10 confirmed important predictors, as shown by the distribution of importance scores in Figure 1A. In parallel, least absolute shrinkage and selection operator regression (Lasso) was fitted, with the coefficient traces displayed in Figure 1B and the selection of the penalty parameter guided by cross validated binomial deviance in Figure 1C. At the optimal penalty, thirteen predictors had nonzero coefficients.

To enhance robustness and reduce redundancy, we defined the final feature set as the intersection of variables retained by both methods. The Venn diagram in Figure 1D shows three variables unique to Boruta, six unique to least absolute shrinkage and selection operator, and seven variables shared by both. These seven consensus predictors were Alcohol consumption, Triglycerides, Diabetes duration, Health insurance type, Fasting blood glucose, Estimated glomerular filtration rate (eGFR), and Exercise frequency. These features were carried forward for model development.

### Model development and performance

**Figure 2:**
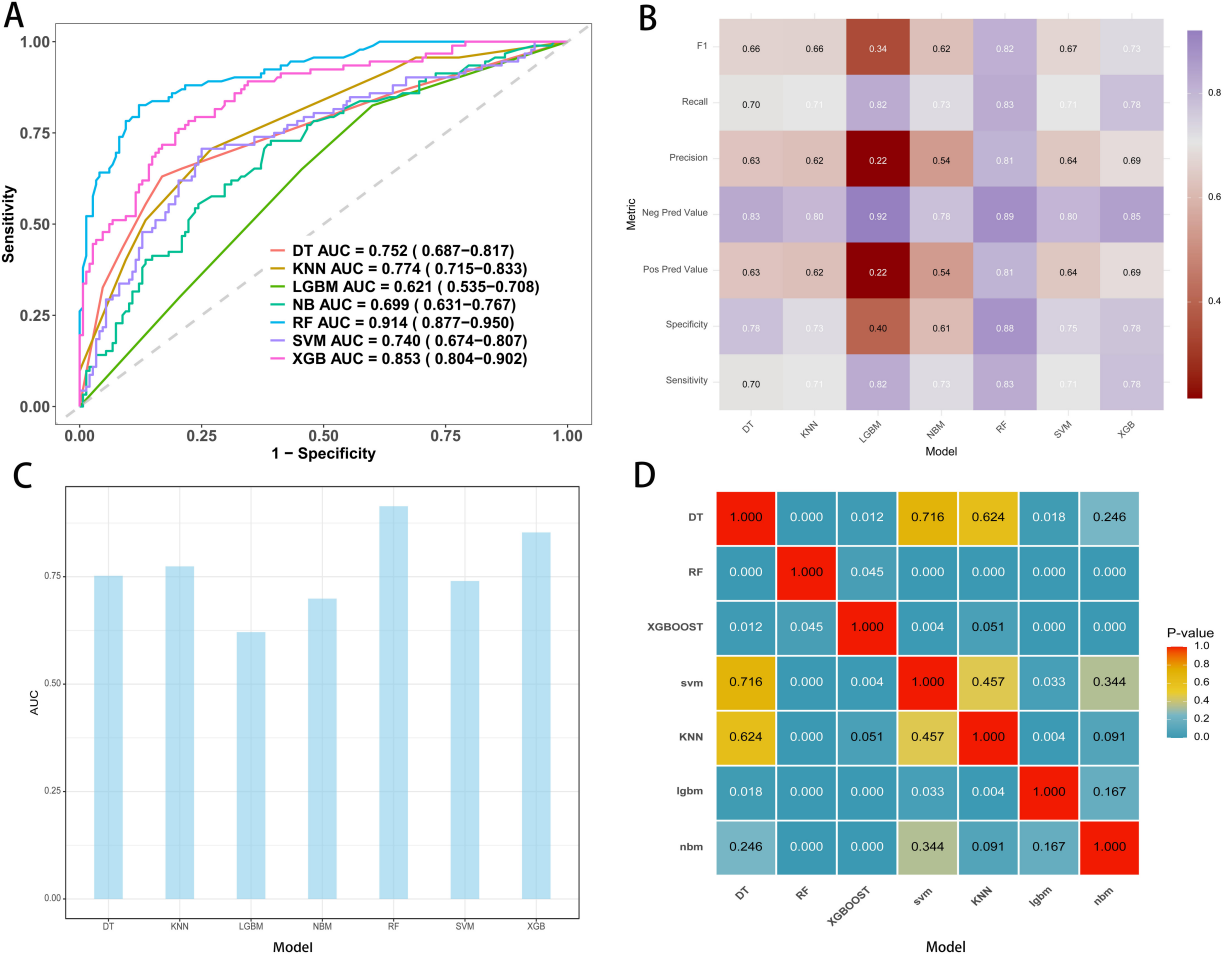
Performance metrics of models in the training cohort. **(A)** ROC curves and AUC values for the seven model. **(B)** Comparative analysis metrics across all seven models. **(C)** AUC display for seven machine learning models. **(D)** Pairwise AUROC comparisons using the DeLong test; cells show *P*-values.

Seven machine learning algorithms were trained on the development cohort using five-fold cross-validation: decision tree (DT), k-nearest neighbors (KNN), light gradient boosting machine (LGBM), naïve Bayes (NB), random forest (RF), support vector machine (SVM), and extreme gradient boosting (XGB). Overall, substantial variation in model discrimination was observed. As illustrated by the receiver operating characteristic (ROC) curves (Figure 2A) and AUC comparison (Figure 2C), the random forest model demonstrated the highest discrimination, with an AUC of 0.914 (95% CI: 0.877–0.950) on the training set. The XGB model also performed well, achieving an AUC of 0.853 (95% CI: 0.804–0.902), while other models such as KNN, DT, and SVM showed moderate predictive ability, with AUCs ranging from 0.74 to 0.77. In contrast, the LGBM model had the lowest AUC at 0.621 (95% CI: 0.535–0.708).

Classifier metrics for each model, including F1 score, recall, precision, negative predictive value (NPV), positive predictive value (PPV), specificity, and sensitivity, are summarized in the heatmap (Figure 2B). The random forest achieved the highest performance across most metrics, including an F1 score of 0.82, specificity of 0.88, and sensitivity of 0.83, indicating balanced accuracy in both identifying positive and negative cases.

Pairwise statistical comparison of AUCs is shown in the *P*-value matrix (Figure 2D). The random forest model significantly outperformed all other algorithms (all *P*-values < 0.05), except for itself, confirming the robustness of its superior predictive performance. Based on these analyses, the random forest model was selected as the final predictive model for subsequent interpretation and external validation.

### Model performance on both the testing and external validation sets

**Figure 3:**
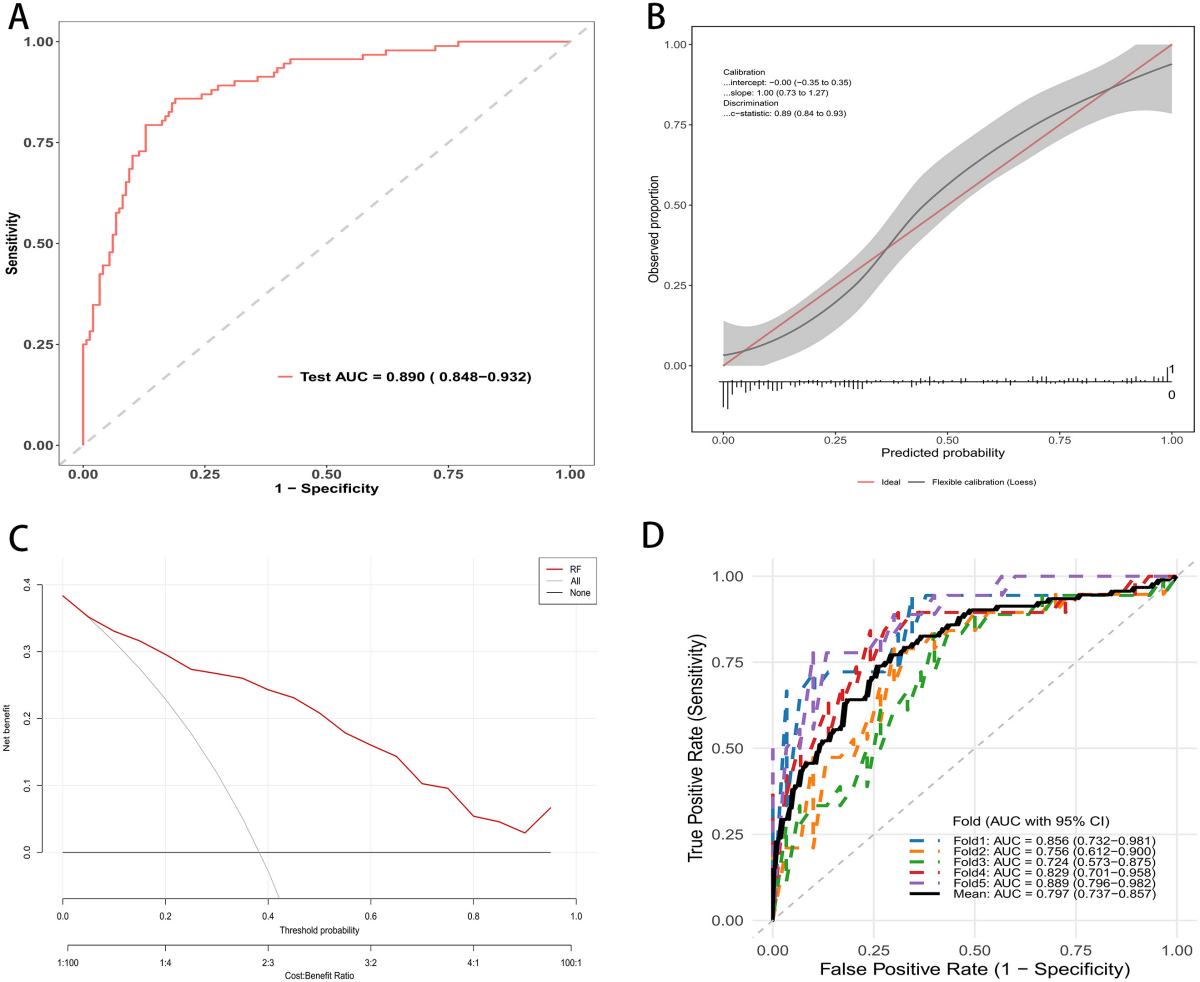
RF mode evaluation in testing cohort. **(A)** ROC curve on the independent test set; AUC = 0.890 (95% CI: 0.848–0.932). **(B)** Calibration plot with flexible curve and 95% CI band vs. the ideal line; calibration intercept, slope, and c-statistic are shown. **(C)** Decision curve analysis: net benefit across threshold probabilities for the random forest (red) compared with treat-all and treat-none strategies. **(D)** Five-fold cross-validated ROC curves in the testing cohort; colored lines show each fold, the thick black line the mean; legend reports fold-specific AUCs and the mean (95% CI).

On the independent testing set, the random forest model demonstrated strong discrimination. The receiver operating characteristic curve showed an AUC of 0.890 with a 95 percent confidence interval of 0.848 to 0.932 (Figure 3A). At the optimal probability threshold, the model achieved a sensitivity of 85.0%, a specificity of 78.0%, a positive predictive value (PPV) of 70.0%, and a negative predictive value (NPV) of 89.0% (Supplementary Table S2). Calibration was satisfactory, with an intercept of approximately 0.00 with a 95 percent confidence interval of −0.35 to 0.35 and a slope of 1.00 with a 95 percent confidence interval of 0.73 to 1.27. The flexible calibration curve closely followed the ideal line, and the c statistic was 0.89 with a 95 percent confidence interval of 0.86 to 0.93 (Figure 3B). Decision curve analysis indicated higher net benefit for the random forest model than for the strategies of treating all or treating none across a broad range of threshold probabilities, supporting its potential clinical utility (Figure 3C). Performance was consistent across resampled testing folds. The fold-specific AUCs were 0.856, 0.756, 0.742, 0.820, and 0.889, yielding a mean AUC of 0.797 with a 95 percent confidence interval of 0.737 to 0.857 (Figure 3D).

**Figure 4:**
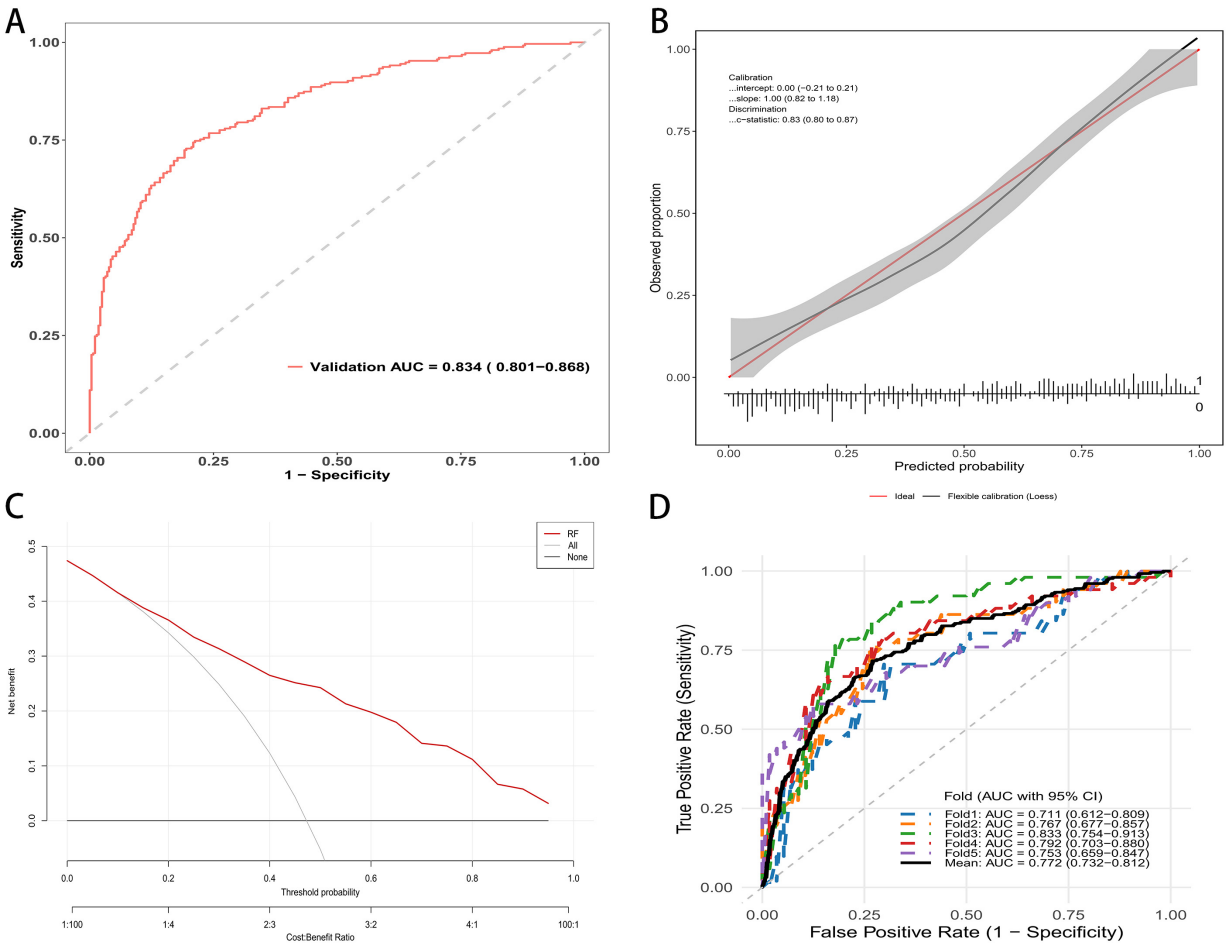
RF mode evaluation in external validation cohort. **(A)** ROC curve in the validation cohort; AUC = 0.834 (95% CI: 0.801–0.868). **(B)** Calibration curve of the external validation set. **(C)** Decision curve analysis: net benefit across threshold probabilities for the model (red) vs. treat-all and treat-none. **(D)** Five-fold cross-validated ROC curves in the validation cohort.

In the external validation dataset, the random forest model retained good discrimination. The receiver operating characteristic curve yielded an AUC of 0.834 with a 95 percent confidence interval of 0.801 to 0.868 (Figure 4A). Applying the same threshold, the external validation cohort showed a sensitivity of 70.0%, a specificity of 90.0%, a PPV of 81.0%, and an NPV of 83.0% (Supplementary Table S2). Calibration was acceptable, with an intercept of 0.00 with a 95 percent confidence interval of −0.21 to 0.21 and a slope of 1.00 with a 95 percent confidence interval of 0.82–1.18. The c statistic was 0.83 with a 95 percent confidence interval of 0.80–0.87 (Figure 4B). Decision curve analysis demonstrated consistent clinical utility, with higher net benefit for the random forest model than for the strategies of treating all or treating none across a wide range of threshold probabilities (Figure 4C). Performance was stable across validation folds. The fold specific AUCs ranged from 0.711 to 0.833, and the mean AUC was 0.772 with a 95 percent confidence interval of 0.732–0.812 (Figure 4D). These findings support the reproducibility and generalizability of the model in an independent population.

### Model interpretability

**Figure 5:**
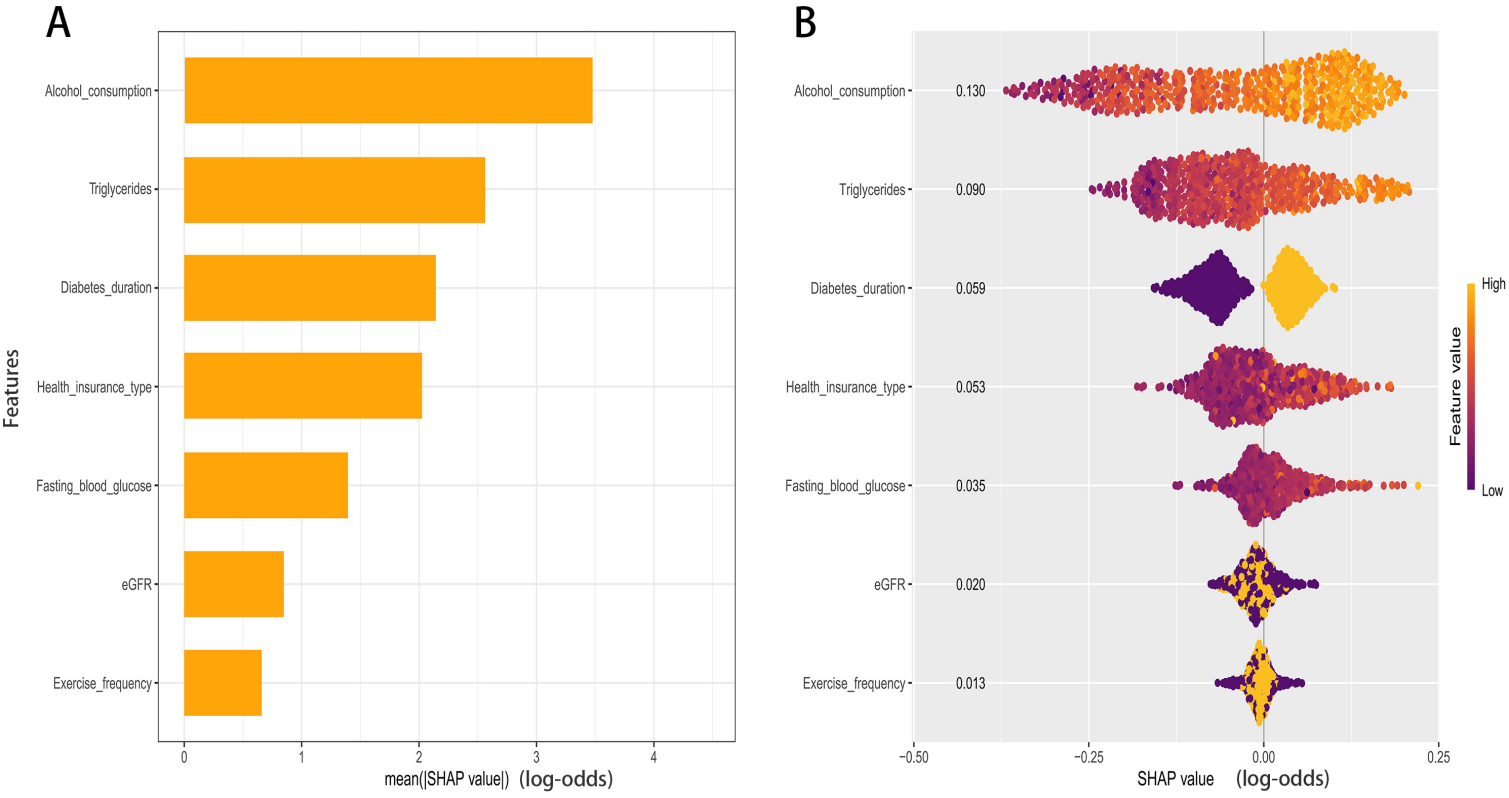
SHAP analysis of the RF model. **(A)** Global importance ranked by mean absolute SHAP value. **(B)** Beeswarm of per-patient SHAP values for key predictors.

Model explanations were generated using SHAP. The global importance plot ranked predictors by their mean absolute SHAP values, indicating that Alcohol consumption contributed most to the predictions, followed by Triglycerides, Diabetes duration, Health insurance type, Fasting blood glucose, estimated glomerular filtration rate (eGFR), and Exercise frequency (Figure 5A).

The color gradient in the summary plot revealed the directions of effect (Figure 5B). Higher levels of alcohol consumption, triglycerides, and fasting blood glucose were associated with higher SHAP values, indicating a greater contribution to the predicted probability of comorbid hypertension. Similarly, a longer duration of diabetes corresponded to increased risk contributions. Conversely, lower values of estimated glomerular filtration rate (eGFR) were associated with higher positive SHAP values. For exercise frequency, higher values generally corresponded to lower SHAP values. The contribution of health insurance type varied across specific categories.

## Discussion

This study developed and externally validated a machine learning model to predict comorbid hypertension in patients with type 2 diabetes using routinely collected clinical data. Among several tested algorithms, the random forest model demonstrated the best performance, with excellent discrimination and calibration in both internal and external cohorts. Its strong performance highlights the ability of ensemble learning to capture nonlinear relationships and complex feature interactions that traditional regression approaches may overlook. The model achieved an area under the curve (AUC) of 0.89 in internal validation and 0.83 in external validation, indicating good generalizability across populations. SHAP-based explainability further clarified the contribution and direction of each predictor, supporting clinical interpretability and potential real-world application. Our study demonstrates the superior performance of machine learning over traditional methods in this context. Previous risk prediction models for hypertension in diabetic populations were predominantly based on logistic regression and reported AUCs ranging from approximately 0.72–0.78. In contrast, our Random Forest model achieved an AUC of 0.890 in the testing set and 0.834 in the external validation set. This improvement is likely attributable to the algorithm's ability to capture complex, non-linear interactions among clinical and lifestyle variables that linear models often miss. Furthermore, unlike many “black-box” machine learning studies, our integration of SHAP values provides granular interpretability comparable to traditional nomograms, bridging the gap between predictive accuracy and clinical transparency.

The selected predictors, including alcohol consumption, triglycerides, diabetes duration, health insurance type, fasting blood glucose, estimated glomerular filtration rate (eGFR), and exercise frequency, represent a coherent combination of metabolic, behavioral, and socioeconomic factors. These findings are consistent with previous research linking poor metabolic control and unhealthy lifestyle behaviors with both the incidence and progression of hypertension in diabetes (9). Elevated triglycerides and fasting glucose reflect insulin resistance and chronic hyperglycemia, which promote oxidative stress and endothelial dysfunction (10, 11). These processes impair nitric oxide bioavailability, increase arterial stiffness, and enhance sympathetic activation, eventually leading to higher blood pressure (12, 13). Similarly, longer diabetes duration and reduced eGFR indicate advanced microvascular and renal injury (14). Glomerulosclerosis, sodium retention, and activation of the renin–angiotensin–aldosterone system (RAAS) may further exacerbate vascular resistance and cause a self-reinforcing cycle between hyperglycemia and hypertension (15, 16). Regular exercise and limited alcohol use appeared protective, which is consistent with existing preventive recommendations and may be mediated by improved insulin sensitivity, reduced inflammation, and better autonomic balance (17–19).

The influence of health insurance type may represent disparities in healthcare access, disease monitoring, or medication adherence (20). Patients with more comprehensive coverage may have better control of blood glucose and lipid levels due to timely interventions and follow-up, which indirectly reduce vascular burden (21, 22). This finding highlights that social and behavioral determinants are essential components of cardiometabolic risk. Integration of these factors into predictive models could improve both accuracy and fairness across patient populations. We acknowledge that this variable is specific to the Chinese healthcare system and structurally different from insurance models in other countries. However, in our study context, insurance type serves as a critical proxy for socioeconomic status (SES) and healthcare accessibility, which are universal determinants of chronic disease management. Therefore, while the specific categorical definitions may not be directly transportable, the underlying predictive value of SES is generalizable. We recommend that future studies attempting to validate or apply this model in international settings substitute this variable with locally relevant SES indicators to maintain the model's predictive performance and ecological validity.

The explainability analysis using SHAP provided additional interpretative power. The SHAP values not only ranked features by their global contribution to prediction but also allowed individualized explanation of risk, supporting model transparency and potential bedside application. The ability to visualize how each variable contributes to patient-specific risk enables clinicians to connect machine learning outputs with known pathophysiological pathways, reinforcing clinical confidence in algorithm-guided decisions. The interpretability provided by SHAP values transforms our model from a predictive tool into a guide for clinical decision-making. Unlike traditional black-box models that output a simple risk score, SHAP visualizations allow clinicians to identify the specific drivers of hypertension risk for each individual patient. For instance, our SHAP analysis highlighted the substantial protective effect of regular exercise. Clinically, this allows physicians to move beyond generic advice and visually demonstrate to patients how increasing physical activity could quantitatively lower their specific risk score. Furthermore, the identification of socioeconomic factors, such as health insurance type, as key predictors suggests that clinical risk assessment should extend beyond biological metrics. For patients with specific insurance types associated with higher risk, which may reflect lower healthcare access or economic constraints, clinicians might consider more frequent blood pressure monitoring or earlier aggressive interventions. Thus, the model supports a precision medicine approach, enabling targeted modification of lifestyle factors and resource allocation based on individual risk profiles.

While several predictive models for hypertension in diabetic patients have been proposed, our study offers distinct advantages in terms of methodology and variable comprehensiveness. First, regarding predictive performance, our Random Forest model achieved an AUC of 0.890 in the internal testing set and 0.834 in external validation. These results outperform many previous models based on conventional logistic regression that typically report AUCs in the range of 0.70–0.80. Second, unlike studies that relied exclusively on traditional clinical metrics such as age, BMI, and blood pressure history, our model integrates multidimensional predictors. These include behavioral factors like alcohol consumption and exercise frequency, and notably, socioeconomic status represented by health insurance type. The inclusion of health insurance type adds a novel layer of risk stratification by reflecting the impact of healthcare access on comorbidity management. Finally, a common criticism of machine learning models is their black-box nature. By implementing SHAP analysis, we provided a transparent explanation of feature contributions, including the protective effect of exercise and the risk associated with specific insurance types. This approach enhances the clinical trustworthiness of our tool compared to non-interpretable algorithms. Given the high accessibility of the predictors used in our random forest model, it is particularly well-suited for implementation in primary care settings. The model relies on routine demographic, lifestyle, and biochemical data, such as age, BMI, and fasting blood glucose, that are standardly collected during regular check-ups, avoiding the need for specialized or costly diagnostics. Practically, this model could be deployed as an automated screening tool integrated into electronic health records (EHR). In this role, it would function as a decision support system, automatically calculating risk scores for patients with type 2 diabetes during routine visits. This would enable general practitioners to stratify patients efficiently and target high-risk individuals for early preventive strategies, such as intensified lifestyle counseling or more frequent blood pressure monitoring, thereby optimizing resource allocation and potentially delaying the onset of hypertension.

Despite these strengths, several limitations should be acknowledged. First, the retrospective data collection from tertiary care centers may introduce selection bias. Patients in these settings often present with more severe disease states or complex comorbidities compared to the general community population, which may limit the generalizability of our findings to primary care settings. Second, the model relied on baseline data measured at a single time point. This cross-sectional approach limits our ability to infer causal relationships or account for dynamic changes in risk factors over time, highlighting the need for future longitudinal studies to validate the model's temporal stability. Third, medication use beyond antihypertensives was not thoroughly analyzed, which may influence metabolic parameters and blood pressure regulation. Finally, the integration of molecular biomarkers, such as inflammatory cytokines or genetic polymorphisms, could further improve predictions and illuminate underlying biological mechanisms in future iterations of the model.

Moving forward, translating this prediction model into clinical practice requires rigorous validation and assessment. The immediate priority is to conduct large-scale prospective studies to confirm the model's performance across diverse populations, ensuring its reliability in different healthcare settings. Beyond validation, clinical impact studies are essential to evaluate the real-world utility of the model. By integrating the risk score into electronic health records as a decision support tool, future research should assess whether its use leads to tangible benefits, such as earlier diagnosis of hypertension, better adherence to lifestyle interventions, and ultimately, a reduction in cardiovascular events among patients with type 2 diabetes.

In summary, this study establishes a robust random forest model for predicting the specific risk of comorbid hypertension in patients with type 2 diabetes mellitus (T2DM). By integrating routine metabolic, behavioral, and demographic indicators, the model assists clinicians in early identification by automatically flagging high-risk individuals who may benefit from intensified monitoring. Furthermore, the model's interpretability allows practitioners to visualize personalized risk drivers, including specific lifestyle factors, which facilitates targeted preventive interventions rather than generic advice. Future work should focus on external validation across diverse multi-center cohorts and the integration of this algorithm into electronic health record systems to evaluate its real-world clinical impact.

## Data Availability

The original contributions presented in the study are included in the article/Supplementary material, further inquiries can be directed to the corresponding author.
