# Supplementary figures and images for: Development and validation of a machine learning model to predict comorbid hypertension in patients with type 2 diabetes
Source: Front Med (Lausanne). 2026 Feb 18;13:1754916. doi: 10.3389/fmed.2026.1754916 (PMC12956727; doi:10.3389/fmed.2026.1754916)

**Figure S1.** Flowchart of the study design and model development process.


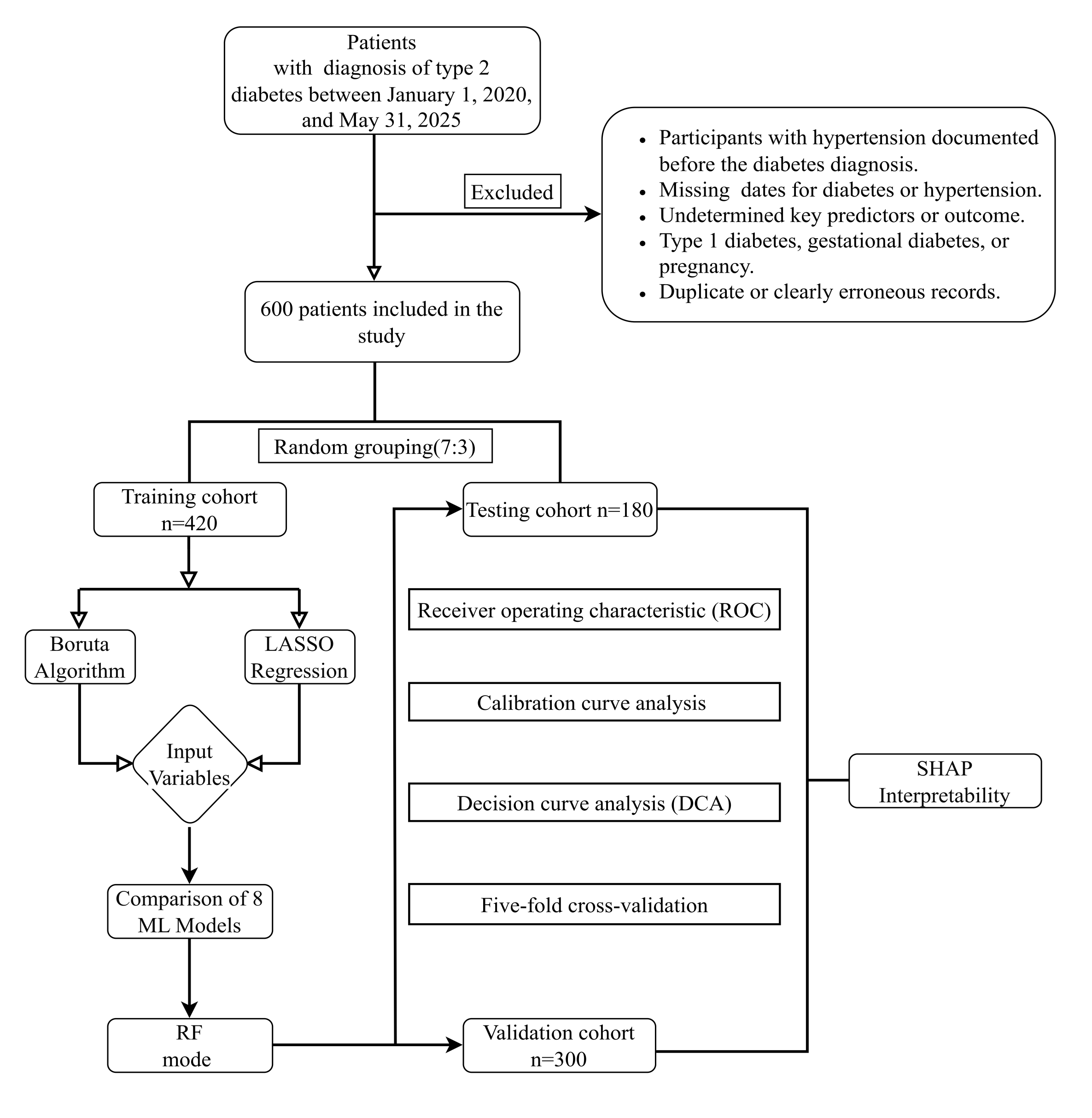

Supplement: Supplementary file 2 [file Supplementary_file_1.docx]
