# Supplementary material for: Development and validation of a machine learning model to predict comorbid hypertension in patients with type 2 diabetes
Source: Front Med (Lausanne). 2026 Feb 18;13:1754916. doi: 10.3389/fmed.2026.1754916 (PMC12956727; doi:10.3389/fmed.2026.1754916)
Supplement: Supplementary file 3 [file Table_1.docx]

**Supplementary Table S1** List of tuned hyperparameters and search ranges for the seven machine learning algorithms.

| **Algorithm** | **Hyperparameter** | **Search Range** |
| --- | --- | --- |
| **Decision Tree (DT)** | max_depth | [3, 5, 7, 10, None] |
|  | min_samples_split | [2, 5, 10] |
|  | min_samples_leaf | [1, 2, 4] |
|  | criterion | ['gini', 'entropy'] |
| **K-Nearest Neighbors (KNN)** | n_neighbors | [3, 5, 7, 9, 11, 15, 20] |
|  | weights | ['uniform', 'distance'] |
|  | p (Metric) | [1, 2] |
| **LightGBM (LGBM)** | learning_rate | [0.01, 0.05, 0.1, 0.2] |
|  | n_estimators | [100, 200, 500, 1000] |
|  | num_leaves | [20, 31, 50, 100] |
|  | max_depth | [-1, 5, 10, 20] |
| **Naïve Bayes (NB)** | var_smoothing | [1e-9, 1e-8, 1e-7, 1e-6] |
| **Random Forest (RF)** | n_estimators | [100, 200, 500, 800] |
|  | max_depth | [5, 10, 20, 30, None] |
|  | min_samples_split | [2, 5, 10] |
|  | max_features | ['sqrt', 'log2'] |
| **Support Vector Machine (SVM)** | C | [0.1, 1, 10, 100, 1000] |
|  | kernel | ['linear', 'rbf'] |
|  | gamma | ['scale', 'auto', 0.01, 0.1] |
| **XGBoost (XGB)** | learning_rate | [0.01, 0.05, 0.1, 0.3] |
|  | n_estimators | [100, 300, 500, 1000] |
|  | max_depth | [3, 5, 6, 9] |
|  | subsample | [0.6, 0.8, 1.0] |
|  | colsample_bytree | [0.6, 0.8, 1.0] |
