# Supplementary material for: Development and validation of a machine learning model to predict comorbid hypertension in patients with type 2 diabetes
Source: Front Med (Lausanne). 2026 Feb 18;13:1754916. doi: 10.3389/fmed.2026.1754916 (PMC12956727; doi:10.3389/fmed.2026.1754916)
Supplement: Supplementary file 4 [file Table_2.docx]

Table S2. Performance metrics of the random forest model in the testing and external validation cohorts at the optimal threshold.

| Dataset | F1 | Recall | Precision | Neg Pred Valve | Pos Pred Value | Specificity | Sensitivity |
| --- | --- | --- | --- | --- | --- | --- | --- |
| Testing cohort | 0.77 | 0.85 | 0.70 | 0.89 | 0.70 | 0.78 | 0.85 |
| Validation cohort | 0.75 | 0.70 | 0.81 | 0.83 | 0.81 | 0.90 | 0.70 |
